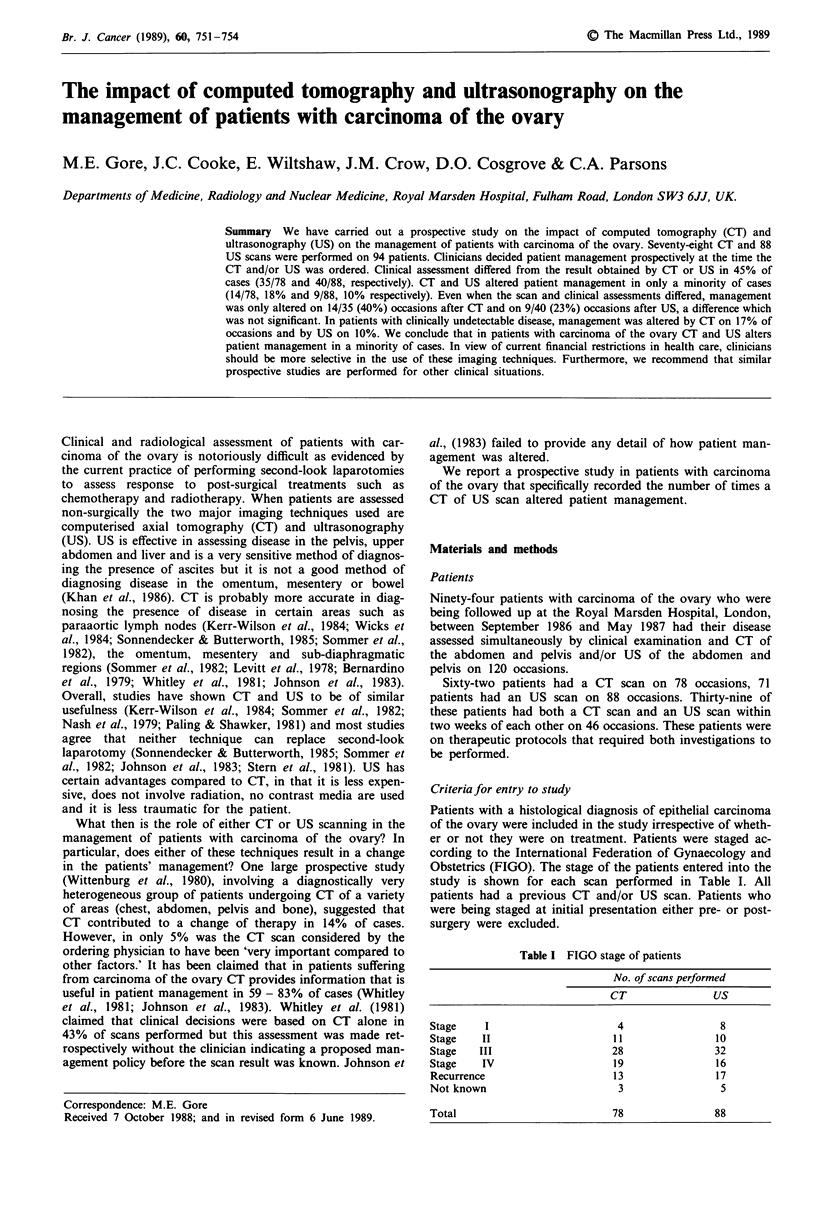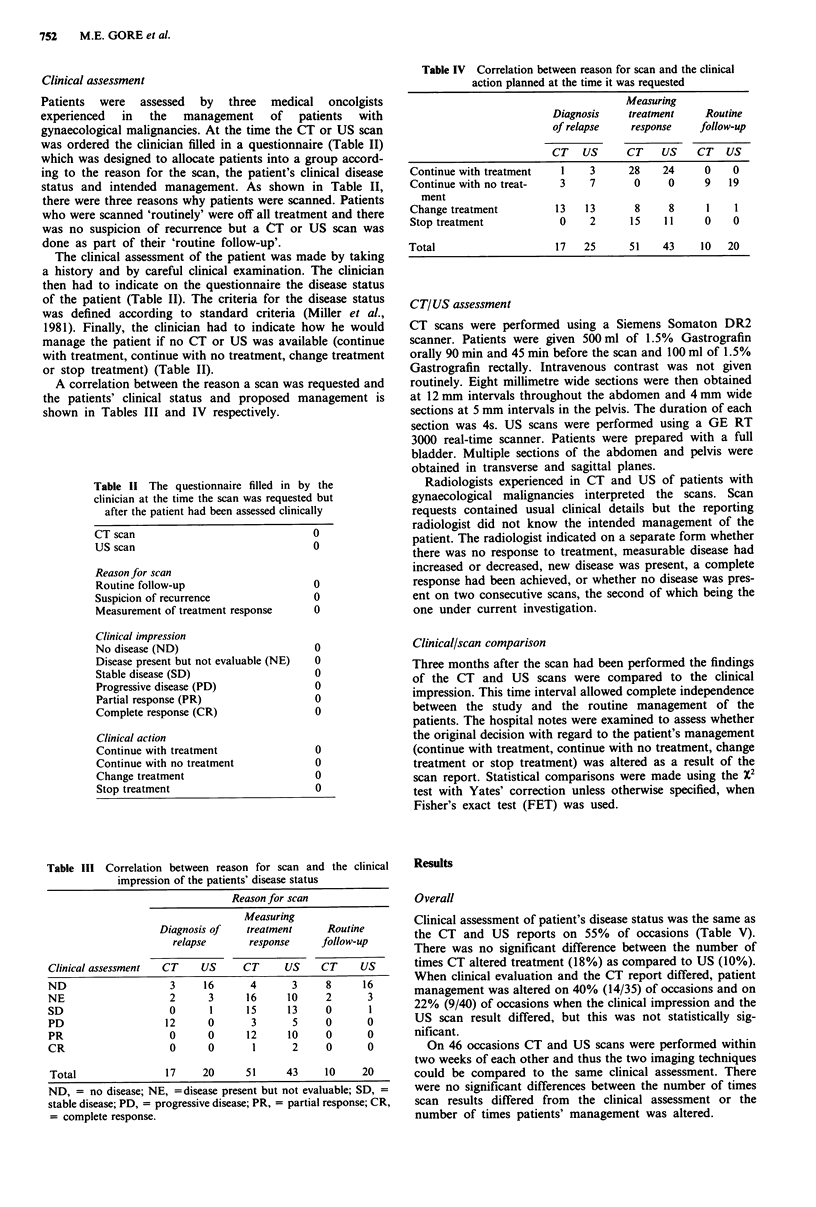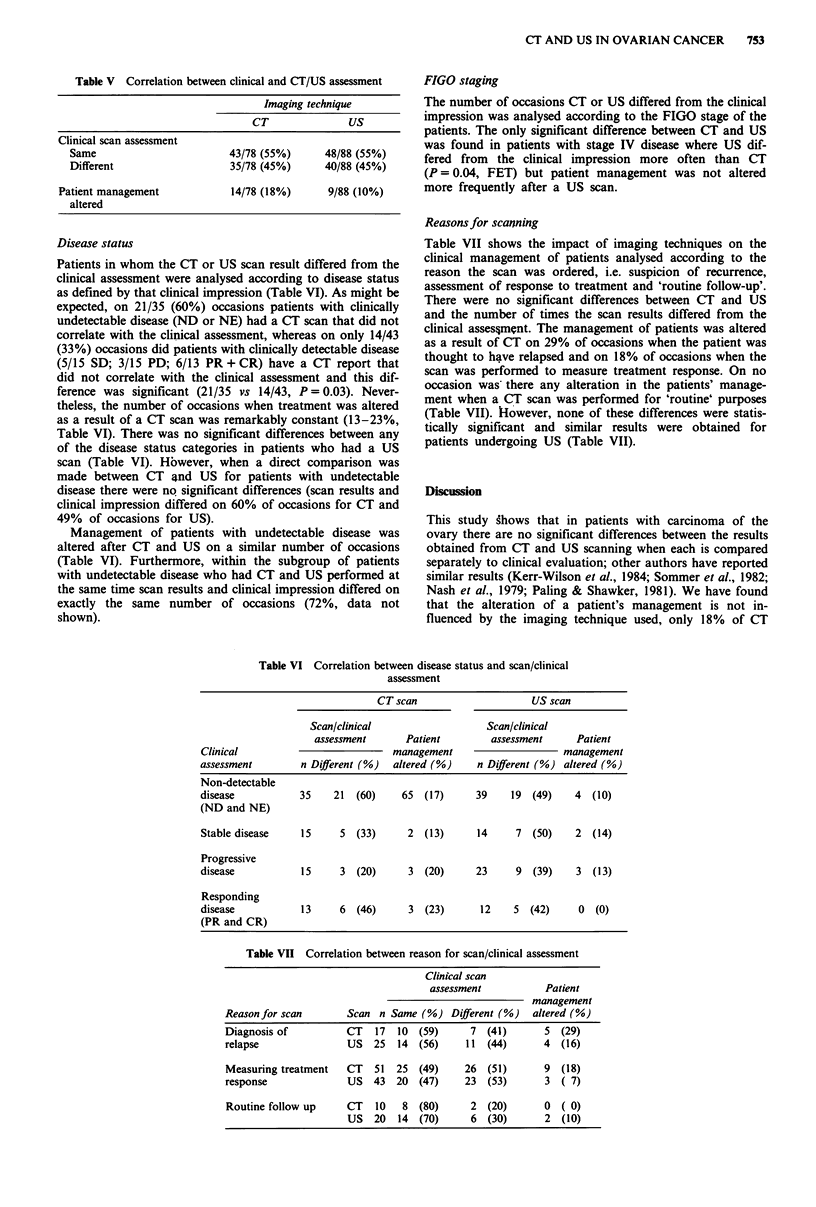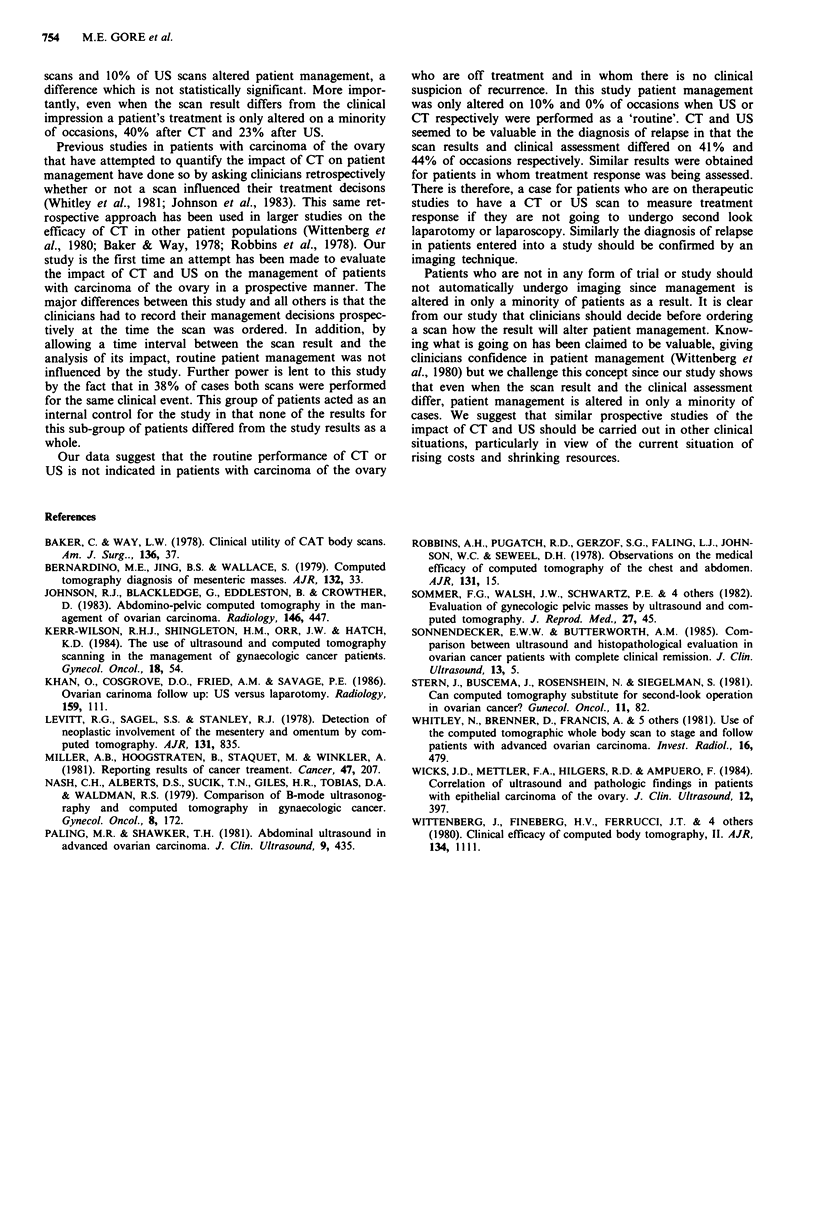# The impact of computed tomography and ultrasonography on the management of patients with carcinoma of the ovary.

**DOI:** 10.1038/bjc.1989.352

**Published:** 1989-11

**Authors:** M. E. Gore, J. C. Cooke, E. Wiltshaw, J. M. Crow, D. O. Cosgrove, C. A. Parsons

**Affiliations:** Department of Medicine, Royal Marsden Hospital, London, UK.

## Abstract

We have carried out a prospective study on the impact of computed tomography (CT) and ultrasonography (US) on the management of patients with carcinoma of the ovary. Seventy-eight CT and 88 US scans were performed on 94 patients. Clinicians decided patient management prospectively at the time the CT and/or US was ordered. Clinical assessment differed from the result obtained by CT or US in 45% of cases (35/78 and 40/88, respectively). CT and US altered patient management in only a minority of cases (14/78, 18% and 9/88, 10% respectively). Even when the scan and clinical assessments differed, management was only altered on 14/35 (40%) occasions after CT and on 9/40 (23%) occasions after US, a difference which was not significant. In patients with clinically undetectable disease, management was altered by CT on 17% of occasions and by US on 10%. We conclude that in patients with carcinoma of the ovary CT and US alters patient management in a minority of cases. In view of current financial restrictions in health care, clinicians should be more selective in the use of these imaging techniques. Furthermore, we recommend that similar prospective studies are performed for other clinical situations.


Br. J. Cancer (1989), 60, 751–754 © The Macmillan Press Ltd., 1989

### The impact of computed tomography and ultrasonography on the management of patients with carcinoma of the ovary

M.E. Gore, J.C. Cooke, E. Wiltshaw, J.M. Crow, D.O. Cosgrove & C.A. Parsons

*Departments of Medicine, Radiology and Nuclear Medicine, Royal Marsden Hospital, Fulham Road, London SW3 6JJ, UK.*

**Summary** We have carried out a prospective study on the impact of computed tomography (CT) and ultrasonography (US) on the management of patients with carcinoma of the ovary. Seventy-eight CT and 88 US scans were performed on 94 patients. Clinicians decided patient management prospectively at the time the CT and/or US was ordered. Clinical assessment differed from the result obtained by CT or US in 45% of cases (35/78 and 40/88, respectively). CT and US altered patient management in only a minority of cases (14/78, 18% and 9/88, 10% respectively). Even when the scan and clinical assessments differed, management was only altered on 14/35 (40%) occasions after CT and on 9/40 (23%) occasions after US, a difference which was not significant. In patients with clinically undetectable disease, management was altered by CT on 17% of occasions and by US on 10%. We conclude that in patients with carcinoma of the ovary CT and US alters patient management in a minority of cases. In view of current financial restrictions in health care, clinicians should be more selective in the use of these imaging techniques. Furthermore, we recommend that similar prospective studies are performed for other clinical situations.

Clinical and radiological assessment of patients with carcinoma of the ovary is notoriously difficult as evidenced by the current practice of performing second-look laparotomies to assess response to post-surgical treatments such as chemotherapy and radiotherapy. When patients are assessed non-surgically the two major imaging techniques used are computerised axial tomography (CT) and ultrasonography (US). US is effective in assessing disease in the pelvis, upper abdomen and liver and is a very sensitive method of diagnosing the presence of ascites but it is not a good method of diagnosing disease in the omentum, mesentery or bowel (Khan *et al.*, 1986). CT is probably more accurate in diagnosing the presence of disease in certain areas such as paraaortic lymph nodes (Kerr-Wilson *et al.*, 1984; Wicks *et al.*, 1984; Sonnendecker & Butterworth, 1985; Sommer *et al.*, 1982), the omentum, mesentery and sub-diaphragmatic regions (Sommer *et al.*, 1982; Levitt *et al.*, 1978; Bernardino *et al.*, 1979; Whitley *et al.*, 1981; Johnson *et al.*, 1983). Overall, studies have shown CT and US to be of similar usefulness (Kerr-Wilson *et al.*, 1984; Sommer *et al.*, 1982; Nash *et al.*, 1979; Paling & Shawker, 1981) and most studies agree that neither technique can replace second-look laparotomy (Sonnendecker & Butterworth, 1985; Sommer *et al.*, 1982; Johnson *et al.*, 1983; Stern *et al.*, 1981). US has certain advantages compared to CT, in that it is less expensive, does not involve radiation, no contrast media are used and it is less traumatic for the patient.

What then is the role of either CT or US scanning in the management of patients with carcinoma of the ovary? In particular, does either of these techniques result in a change in the patients' management? One large prospective study (Wittenburg *et al.*, 1980), involving a diagnostically very heterogeneous group of patients undergoing CT of a variety of areas (chest, abdomen, pelvis and bone), suggested that CT contributed to a change of therapy in 14% of cases. However, in only 5% was the CT scan considered by the ordering physician to have been 'very important compared to other factors.' It has been claimed that in patients suffering from carcinoma of the ovary CT provides information that is useful in patient management in 59 – 83% of cases (Whitley *et al.*, 1981; Johnson *et al.*, 1983). Whitley *et al.* (1981) claimed that clinical decisions were based on CT alone in 43% of scans performed but this assessment was made retrospectively without the clinician indicating a proposed management policy before the scan result was known. Johnson *et al.*, (1983) failed to provide any detail of how patient management was altered.

We report a prospective study in patients with carcinoma of the ovary that specifically recorded the number of times a CT of US scan altered patient management.

### Materials and methods

### *Patients*

Ninety-four patients with carcinoma of the ovary who were being followed up at the Royal Marsden Hospital, London, between September 1986 and May 1987 had their disease assessed simultaneously by clinical examination and CT of the abdomen and pelvis and/or US of the abdomen and pelvis on 120 occasions.

Sixty-two patients had a CT scan on 78 occasions, 71 patients had an US scan on 88 occasions. Thirty-nine of these patients had both a CT scan and an US scan within two weeks of each other on 46 occasions. These patients were on therapeutic protocols that required both investigations to be performed.

### *Criteria for entry to study*

Patients with a histological diagnosis of epithelial carcinoma of the ovary were included in the study irrespective of whether or not they were on treatment. Patients were staged according to the International Federation of Gynaecology and Obstetrics (FIGO). The stage of the patients entered into the study is shown for each scan performed in Table I. All patients had a previous CT and/or US scan. Patients who were being staged at initial presentation either pre- or post-surgery were excluded.

**Table I** FIGO stage of patients

| | *No. of scans performed* | |
|---|---|---|
| | *CT* | *US* |
| Stage I | 4 | 8 |
| Stage II | 11 | 10 |
| Stage III | 28 | 32 |
| Stage IV | 19 | 16 |
| Recurrence | 13 | 17 |
| Not known | 3 | 5 |
| Total | 78 | 88 |

Correspondence: M.E. Gore
Received 7 October 1988; and in revised form 6 June 1989.

*Clinical assessment*

Patients were assessed by three medical oncolgists experienced in the management of patients with gynaecological malignancies. At the time the CT or US scan was ordered the clinician filled in a questionnaire (Table II) which was designed to allocate patients into a group according to the reason for the scan, the patient's clinical disease status and intended management. As shown in Table II, there were three reasons why patients were scanned. Patients who were scanned 'routinely' were off all treatment and there was no suspicion of recurrence but a CT or US scan was done as part of their 'routine follow-up'.

The clinical assessment of the patient was made by taking a history and by careful clinical examination. The clinician then had to indicate on the questionnaire the disease status of the patient (Table II). The criteria for the disease status was defined according to standard criteria (Miller *et al.*, 1981). Finally, the clinician had to indicate how he would manage the patient if no CT or US was available (continue with treatment, continue with no treatment, change treatment or stop treatment) (Table II).

A correlation between the reason a scan was requested and the patients' clinical status and proposed management is shown in Tables III and IV respectively.

**Table II** The questionnaire filled in by the clinician at the time the scan was requested but after the patient had been assessed clinically

| | |
|---|---|
| CT scan | 0 |
| US scan | 0 |
| *Reason for scan* | |
| Routine follow-up | 0 |
| Suspicion of recurrence | 0 |
| Measurement of treatment response | 0 |
| *Clinical impression* | |
| No disease (ND) | 0 |
| Disease present but not evaluable (NE) | 0 |
| Stable disease (SD) | 0 |
| Progressive disease (PD) | 0 |
| Partial response (PR) | 0 |
| Complete response (CR) | 0 |
| *Clinical action* | |
| Continue with treatment | 0 |
| Continue with no treatment | 0 |
| Change treatment | 0 |
| Stop treatment | 0 |

**Table III** Correlation between reason for scan and the clinical impression of the patients' disease status

| | *Reason for scan* | | | | | |
|---|---|---|---|---|---|---|
| | *Diagnosis of relapse* | | *Measuring treatment response* | | *Routine follow-up* | |
| *Clinical assessment* | *CT* | *US* | *CT* | *US* | *CT* | *US* |
| ND | 3 | 16 | 4 | 3 | 8 | 16 |
| NE | 2 | 3 | 16 | 10 | 2 | 3 |
| SD | 0 | 1 | 15 | 13 | 0 | 1 |
| PD | 12 | 0 | 3 | 5 | 0 | 0 |
| PR | 0 | 0 | 12 | 10 | 0 | 0 |
| CR | 0 | 0 | 1 | 2 | 0 | 0 |
| Total | 17 | 20 | 51 | 43 | 10 | 20 |

ND, = no disease; NE, =disease present but not evaluable; SD, = stable disease; PD, = progressive disease; PR, = partial response; CR, = complete response.

**Table IV** Correlation between reason for scan and the clinical action planned at the time it was requested

| | *Diagnosis of relapse* | | *Measuring treatment response* | | *Routine follow-up* | |
|---|---|---|---|---|---|---|
| | *CT* | *US* | *CT* | *US* | *CT* | *US* |
| Continue with treatment | 1 | 3 | 28 | 24 | 0 | 0 |
| Continue with no treatment | 3 | 7 | 0 | 0 | 9 | 19 |
| Change treatment | 13 | 13 | 8 | 8 | 1 | 1 |
| Stop treatment | 0 | 2 | 15 | 11 | 0 | 0 |
| Total | 17 | 25 | 51 | 43 | 10 | 20 |

*CT/US assessment*

CT scans were performed using a Siemens Somaton DR2 scanner. Patients were given 500 ml of 1.5% Gastrografin orally 90 min and 45 min before the scan and 100 ml of 1.5% Gastrografin rectally. Intravenous contrast was not given routinely. Eight millimetre wide sections were then obtained at 12 mm intervals throughout the abdomen and 4 mm wide sections at 5 mm intervals in the pelvis. The duration of each section was 4s. US scans were performed using a GE RT 3000 real-time scanner. Patients were prepared with a full bladder. Multiple sections of the abdomen and pelvis were obtained in transverse and sagittal planes.

Radiologists experienced in CT and US of patients with gynaecological malignancies interpreted the scans. Scan requests contained usual clinical details but the reporting radiologist did not know the intended management of the patient. The radiologist indicated on a separate form whether there was no response to treatment, measurable disease had increased or decreased, new disease was present, a complete response had been achieved, or whether no disease was present on two consecutive scans, the second of which being the one under current investigation.

*Clinical/scan comparison*

Three months after the scan had been performed the findings of the CT and US scans were compared to the clinical impression. This time interval allowed complete independence between the study and the routine management of the patients. The hospital notes were examined to assess whether the original decision with regard to the patient's management (continue with treatment, continue with no treatment, change treatment or stop treatment) was altered as a result of the scan report. Statistical comparisons were made using the $\chi^2$ test with Yates' correction unless otherwise specified, when Fisher's exact test (FET) was used.

### Results

*Overall*

Clinical assessment of patient's disease status was the same as the CT and US reports on 55% of occasions (Table V). There was no significant difference between the number of times CT altered treatment (18%) as compared to US (10%). When clinical evaluation and the CT report differed, patient management was altered on 40% (14/35) of occasions and on 22% (9/40) of occasions when the clinical impression and the US scan result differed, but this was not statistically significant.

On 46 occasions CT and US scans were performed within two weeks of each other and thus the two imaging techniques could be compared to the same clinical assessment. There were no significant differences between the number of times scan results differed from the clinical assessment or the number of times patients' management was altered.

**Table V** Correlation between clinical and CT/US assessment

| | Imaging technique | |
|---|---|---|
| | *CT* | *US* |
| Clinical scan assessment | | |
| Same | 43/78 (55%) | 48/88 (55%) |
| Different | 35/78 (45%) | 40/88 (45%) |
| Patient management altered | 14/78 (18%) | 9/88 (10%) |

### *Disease status*

Patients in whom the CT or US scan result differed from the clinical assessment were analysed according to disease status as defined by that clinical impression (Table VI). As might be expected, on 21/35 (60%) occasions patients with clinically undetectable disease (ND or NE) had a CT scan that did not correlate with the clinical assessment, whereas on only 14/43 (33%) occasions did patients with clinically detectable disease (5/15 SD; 3/15 PD; 6/13 PR + CR) have a CT report that did not correlate with the clinical assessment and this difference was significant (21/35 *vs* 14/43, $P = 0.03$). Nevertheless, the number of occasions when treatment was altered as a result of a CT scan was remarkably constant (13–23%, Table VI). There was no significant differences between any of the disease status categories in patients who had a US scan (Table VI). However, when a direct comparison was made between CT and US for patients with undetectable disease there were no significant differences (scan results and clinical impression differed on 60% of occasions for CT and 49% of occasions for US).

Management of patients with undetectable disease was altered after CT and US on a similar number of occasions (Table VI). Furthermore, within the subgroup of patients with undetectable disease who had CT and US performed at the same time scan results and clinical impression differed on exactly the same number of occasions (72%, data not shown).

### *FIGO staging*

The number of occasions CT or US differed from the clinical impression was analysed according to the FIGO stage of the patients. The only significant difference between CT and US was found in patients with stage IV disease where US differed from the clinical impression more often than CT ($P = 0.04$, FET) but patient management was not altered more frequently after a US scan.

### *Reasons for scanning*

Table VII shows the impact of imaging techniques on the clinical management of patients analysed according to the reason the scan was ordered, i.e. suspicion of recurrence, assessment of response to treatment and 'routine follow-up'. There were no significant differences between CT and US and the number of times the scan results differed from the clinical assessment. The management of patients was altered as a result of CT on 29% of occasions when the patient was thought to have relapsed and on 18% of occasions when the scan was performed to measure treatment response. On no occasion was there any alteration in the patients' management when a CT scan was performed for 'routine' purposes (Table VII). However, none of these differences were statistically significant and similar results were obtained for patients undergoing US (Table VII).

### Discussion

This study shows that in patients with carcinoma of the ovary there are no significant differences between the results obtained from CT and US scanning when each is compared separately to clinical evaluation; other authors have reported similar results (Kerr-Wilson *et al.*, 1984; Sommer *et al.*, 1982; Nash *et al.*, 1979; Paling & Shawker, 1981). We have found that the alteration of a patient's management is not influenced by the imaging technique used, only 18% of CT

**Table VI** Correlation between disease status and scan/clinical assessment

| | CT scan | | | US scan | | |
|---|---|---|---|---|---|---|
| | Scan/clinical assessment | | | Scan/clinical assessment | | |
| *Clinical assessment* | *n* | *Different (%)* | *Patient management altered (%)* | *n* | *Different (%)* | *Patient management altered (%)* |
| Non-detectable disease (ND and NE) | 35 | 21 (60) | 65 (17) | 39 | 19 (49) | 4 (10) |
| Stable disease | 15 | 5 (33) | 2 (13) | 14 | 7 (50) | 2 (14) |
| Progressive disease | 15 | 3 (20) | 3 (20) | 23 | 9 (39) | 3 (13) |
| Responding disease (PR and CR) | 13 | 6 (46) | 3 (23) | 12 | 5 (42) | 0 (0) |

**Table VII** Correlation between reason for scan/clinical assessment

| | | | Clinical scan assessment | | |
|---|---|---|---|---|---|
| *Reason for scan* | *Scan* | *n* | *Same (%)* | *Different (%)* | *Patient management altered (%)* |
| Diagnosis of relapse | CT | 17 | 10 (59) | 7 (41) | 5 (29) |
| | US | 25 | 14 (56) | 11 (44) | 4 (16) |
| Measuring treatment response | CT | 51 | 25 (49) | 26 (51) | 9 (18) |
| | US | 43 | 20 (47) | 23 (53) | 3 ( 7) |
| Routine follow up | CT | 10 | 8 (80) | 2 (20) | 0 ( 0) |
| | US | 20 | 14 (70) | 6 (30) | 2 (10) |

scans and 10% of US scans altered patient management, a difference which is not statistically significant. More importantly, even when the scan result differs from the clinical impression a patient's treatment is only altered on a minority of occasions, 40% after CT and 23% after US.

Previous studies in patients with carcinoma of the ovary that have attempted to quantify the impact of CT on patient management have done so by asking clinicians retrospectively whether or not a scan influenced their treatment decisons (Whitley *et al.*, 1981; Johnson *et al.*, 1983). This same retrospective approach has been used in larger studies on the efficacy of CT in other patient populations (Wittenberg *et al.*, 1980; Baker & Way, 1978; Robbins *et al.*, 1978). Our study is the first time an attempt has been made to evaluate the impact of CT and US on the management of patients with carcinoma of the ovary in a prospective manner. The major differences between this study and all others is that the clinicians had to record their management decisions prospectively at the time the scan was ordered. In addition, by allowing a time interval between the scan result and the analysis of its impact, routine patient management was not influenced by the study. Further power is lent to this study by the fact that in 38% of cases both scans were performed for the same clinical event. This group of patients acted as an internal control for the study in that none of the results for this sub-group of patients differed from the study results as a whole.

Our data suggest that the routine performance of CT or US is not indicated in patients with carcinoma of the ovary who are off treatment and in whom there is no clinical suspicion of recurrence. In this study patient management was only altered on 10% and 0% of occasions when US or CT respectively were performed as a 'routine'. CT and US seemed to be valuable in the diagnosis of relapse in that the scan results and clinical assessment differed on 41% and 44% of occasions respectively. Similar results were obtained for patients in whom treatment response was being assessed. There is therefore, a case for patients who are on therapeutic studies to have a CT or US scan to measure treatment response if they are not going to undergo second look laparotomy or laparoscopy. Similarly the diagnosis of relapse in patients entered into a study should be confirmed by an imaging technique.

Patients who are not in any form of trial or study should not automatically undergo imaging since management is altered in only a minority of patients as a result. It is clear from our study that clinicians should decide before ordering a scan how the result will alter patient management. Knowing what is going on has been claimed to be valuable, giving clinicians confidence in patient management (Wittenberg *et al.*, 1980) but we challenge this concept since our study shows that even when the scan result and the clinical assessment differ, patient management is altered in only a minority of cases. We suggest that similar prospective studies of the impact of CT and US should be carried out in other clinical situations, particularly in view of the current situation of rising costs and shrinking resources.

### References

BAKER, C. & WAY, L.W. (1978). Clinical utility of CAT body scans. *Am. J. Surg..*, **136,** 37.

BERNARDINO, M.E., JING, B.S. & WALLACE, S. (1979). Computed tomography diagnosis of mesenteric masses. *AJR*, **132,** 33.

JOHNSON, R.J., BLACKLEDGE, G., EDDLESTON, B. & CROWTHER, D. (1983). Abdomino-pelvic computed tomography in the management of ovarian carcinoma. *Radiology*, **146,** 447.

KERR-WILSON, R.H.J., SHINGLETON, H.M., ORR, J.W. & HATCH, K.D. (1984). The use of ultrasound and computed tomography scanning in the management of gynaecologic cancer patients. *Gynecol. Oncol.*, **18,** 54.

KHAN, O., COSGROVE, D.O., FRIED, A.M. & SAVAGE, P.E. (1986). Ovarian carinoma follow up: US versus laparotomy. *Radiology*, **159,** 111.

LEVITT, R.G., SAGEL, S.S. & STANLEY, R.J. (1978). Detection of neoplastic involvement of the mesentery and omentum by computed tomography. *AJR*, **131,** 835.

MILLER, A.B., HOOGSTRATEN, B., STAQUET, M. & WINKLER, A. (1981). Reporting results of cancer treament. *Cancer*, **47,** 207.

NASH, C.H., ALBERTS, D.S., SUCIK, T.N., GILES, H.R., TOBIAS, D.A. & WALDMAN, R.S. (1979). Comparison of B-mode ultrasonography and computed tomography in gynaecologic cancer. *Gynecol. Oncol.*, **8,** 172.

PALING, M.R. & SHAWKER, T.H. (1981). Abdominal ultrasound in advanced ovarian carcinoma. *J. Clin. Ultrasound*, **9,** 435.

ROBBINS, A.H., PUGATCH, R.D., GERZOF, S.G., FALING, L.J., JOHNSON, W.C. & SEWEEL, D.H. (1978). Observations on the medical efficacy of computed tomography of the chest and abdomen. *AJR*, **131,** 15.

SOMMER, F.G., WALSH, J.W., SCHWARTZ, P.E. & 4 others (1982). Evaluation of gynecologic pelvic masses by ultrasound and computed tomography. *J. Reprod. Med.*, **27,** 45.

SONNENDECKER, E.W.W. & BUTTERWORTH, A.M. (1985). Comparison between ultrasound and histopathological evaluation in ovarian cancer patients with complete clinical remission. *J. Clin. Ultrasound*, **13,** 5.

STERN, J., BUSCEMA, J., ROSENSHEIN, N. & SIEGELMAN, S. (1981). Can computed tomography substitute for second-look operation in ovarian cancer? *Gunecol. Oncol.*, **11,** 82.

WHITLEY, N., BRENNER, D., FRANCIS, A. & 5 others (1981). Use of the computed tomographic whole body scan to stage and follow patients with advanced ovarian carcinoma. *Invest. Radiol.*, **16,** 479.

WICKS, J.D., METTLER, F.A., HILGERS, R.D. & AMPUERO, F. (1984). Correlation of ultrasound and pathologic findings in patients with epithelial carcinoma of the ovary. *J. Clin. Ultrasound*, **12,** 397.

WITTENBERG, J., FINEBERG, H.V., FERRUCCI, J.T. & 4 others (1980). Clinical efficacy of computed body tomography, II. *AJR*, **134,** 1111.